# Association of the rs562556 PCSK9 Gene Polymorphism with Reduced Mortality in Severe Malaria among Malian Children

**DOI:** 10.1155/2020/9340480

**Published:** 2020-09-16

**Authors:** Olesya Fedoryak, Charles Arama, Issa Diarra, Bouréma Kouriba, Michel Chrétien, Majambu Mbikay

**Affiliations:** ^1^Functional Endoproteolysis Laboratory, Clinical Research Institute of Montreal, Montreal H2W 1R7, Quebec, Canada; ^2^Department of Chemistry, Faculty of Sciences, University of Manitoba, Winnipeg R3T 2N2, Manitoba, Canada; ^3^Malaria Research and Training Center, Department of Epidemiology of Parasitic Diseases, International Center of Excellence in Research, University of Sciences, Technique and Technology of Bamako, Bamako, Mali; ^4^Chronic Disease Program, Ottawa Hospital Research Institute, Ottawa K1H 8L6, Ontario, Canada

## Abstract

Recent evidence suggests that proprotein convertase subtilisin/kexin type 9 (PCSK9), a downmodulator of cellular uptake of blood cholesterol, also negatively impacts host immune response to microbial infection. In this study, we investigated whether carrying the loss-of-function (LOF) rs562556 (c.1420 A > G; p.I474 V) PCSK9 single nucleotide polymorphism (SNP) affected the outcome of severe malaria in children. Archival DNA of a cohort of 207 Malian children suffering from severe malaria was genotyped for the rs562556 SNP. Sixty-four children were either heterozygous or homozygous for the minor G allele (carriers); 143 children were homozygous for the common A allele (noncarriers). Among carriers, there was one mortality case (1.6%), compared to 15 cases (10.5%) among noncarriers (*p*=0.0251), suggesting that the G allele is associated with better survival in severe malaria. Intriguingly, this allele did not negatively segregate with any of the clinical symptoms linked to mortality in this cohort. Studies are needed to determine whether PCSK9 inactivation promotes a protective immune response to malaria infection.

## 1. Introduction

Proprotein convertase subtilisin/kexin type 9 (PCSK9) is a liver-secreted protein that binds the low-density lipoprotein receptor (LDLR) at the surface of hepatocytes and escorts it inside the cells towards lysosomes where it gets degraded. It thus reduces LDLR-mediated hepatic uptake of plasma LDL-cholesterol (LDL-C), causing its accumulation in the bloodstream. Single nucleotide polymorphisms (SNPs) in the human PCSK9 gene have been associated with either hypercholesterolemia or hypocholesterolemia, depending on whether they increase (gain-of-function, GOF) or reduce (loss-of-function, LOF) PCSK9 activity [1]. Recent evidence suggests that PCSK9 can modulate the immune response to infection, favorably when less active and deleteriously when overactive [2].

We wanted to verify whether PCSK9 could influence the course of malaria infection. One step to that end is to determine how common GOF and LOF genetic variations segregate with the severity and the outcome of the disease. We previously reported an association of the GOF PCSK9 SNP rs505151 (c.2009 A > G, p.E670 G) with severe malaria in Malian children [3]. Using the same cohort, here we investigate the possible association the LOF SNP rs562556 (c.1420 A > G, p.I474 V) with malaria mortality.

## 2. Subjects and Methods

### 2.1. Study Subjects

Subjects of the present study were 3-month to 14-year-old children diagnosed with severe malaria. They were participants in a previous case-control study investigating physiological factors that increase or decrease the risk for severe malaria [4, 5]. The participants (*N* = 753) were recruited from June 1999 to December 2003 in and around Bandiagara, a circumscription located 700 km east of Bamako, the capital city of Mali. Each case of severe malaria was paired with a case of uncomplicated malaria as well as with a healthy control of the same gender, age, and ethnicity. As per the criteria specified by the World Health Organization [6], severe malaria was defined as a positive *P. falciparum* parasitemia combined with one or several of the following symptoms: impaired consciousness, prostration, multiple convulsions, acidosis, hypoglycemia, renal impairment, severe anemia, jaundice, pulmonary edema, shock, or hyperparasitemia. Inclusion criteria included written informed consent by a participant's adult surrogate, residency in Bandiagara region, and availability for the study duration. Exclusion criteria included participation in a clinical interventional trial, long-term usage of medication with antimalarial activity, known chronic disease or immunosuppressive condition, and any condition which could endanger the participant's safety and infringe his/her rights. All severe malaria cases were treated according to local clinical practice guidelines. The treatment outcome, survival or death, was recorded.

### 2.2. Ethics

The Review Board of the Faculty of Medicine, Pharmacy and Dentistry of the University of Mali, and the Research Ethics Committee of the Montreal Clinical Research Institute of Montreal reviewed and approved the study protocol. At recruitment, children's parents or guardians were asked to provide written informed consent.

### 2.3. Genetic Analysis

Blood was collected at recruitment. Drops of it were spotted on Whatman FTA cards, dried, and stored at room temperature until use. Leucocyte genomic DNA was extracted as follows: a bloody paper disc, 6 mm in diameter, was punched out of an FTA card and placed into a tube containing 0.4 ml of phosphate buffered saline, pH 7.4; after a 20 min incubation at room temperature, the paper was pulled down the tube by centrifugation, the supernatant was discarded, and the paper was soaked in 40 *μ*l of a 10 mM NaOH/200 mM NaCl/0.05% sodium dodecyl sulfate solution and heated for 6 min at 95˚C; it was pulled down as above, and the supernatant was collected.

An aliquot of the supernatant was diluted 12.5-fold in water and used in a TaqMan SNP genotyping assay using a Stratagene Mx 3005P thermocycler instrument (Cedar Creek, TX). Briefly, an rs562556 SNP-containing DNA fragment was amplified by polymerase chain reaction (PCR); two allele-specific oligonucleotide probes, each conjugated in 5′ to a distinct fluorochrome and in 3′ to a quencher, were allowed to anneal to the PCR amplicon, while concomitant removal of the 3′ quencher generated signature allele-specific fluorescence [7]. The supplementary file describes the rs562556 SNP sequence context, fluorogenic probes, and the PCR protocol.

### 2.4. Statistical Analysis

Using GraphPad Prism 8.4.3 software package, Fisher' exact tests were applied to compare the frequencies of death/survival outcome or of severe malaria symptoms between carriers and noncarriers of the minor G allele. The strength of genotypic association was expressed as either odds ratio (OR) or relative risk (RR) and 95% confidence intervals (95% CI). A *P* value of <0.05 was set for significance in all analyses.

## 3. Results

Of 207 children diagnosed with severe malaria, 16 (7.7%) died and 191 (92.3%) survived. As shown in Supplementary Table 1S, age, gender, ethnicity, medication, and use of antimosquito protection were similar between the two groups. However, in survivors, glycemia and hemoglobinemia were higher, while the white blood cell count was lower.

The distribution of individuals symptoms between the groups and their associations with the outcomes are described in Table 1. Mortality in this cohort was significantly associated with respiratory distress (*p* < 0.0001), coma (*p* < 0.001), severe anemia and repeated vomiting (*p* < 0.005), lethargy (*p* < 0.01), convulsion, and prostration and hypoglycemia (*p* < 0.05). Hyperparasitemia (≥5 × 10^5^ parasites/*µ*L), was associated with lower mortality (*p* < 0.005) in this cohort.

We examined whether mortality to severe malaria occurred differentially between patients who carried the LOF G allele of the rs562556 PCSK9 SNP (AG and GG genotypes) and those who did not (AA genotype). As shown in Table 2, among the 64 carriers, one mortality (1.6%) was recorded, compared to 15 mortalities (10.5%) among the 143 of noncarriers, giving an RR (95% CI) of 6.713 (1.19–39.53) (*p*=0.0251), suggesting that the G allele is associated with lower fatality in severe malaria.

Surprisingly, none of the symptoms associated with mortality segregated differentially between the two genotypic groups (Table 3), making it difficult to explain the apparent link of the G allele to better survival. On the other hand, abdominal pain was significantly more frequent in carriers (23/64 (35.9%)) than in noncarriers (16/143 (11.2%)) (*p* < 0.0001).

## 4. Discussion

Before exploring the association of the rs562556 PCSK9 SNP with malaria mortality, we determined the clinical predictors of fatal malaria in this Malian cohort. Except for severe anemia, they corresponded to those identified in a meta-analysis of a large number of studies on African children [8]. Hyperparasitemia and severe anemia have been previously found not to be associated with malaria fatality in Gabonese children [9].

With a minor allele frequency of 0.203, the rs562556 PCSK9 SNP was relatively common in this cohort. A meta-analysis of previous genetic surveys of populations of different ancestries has established that the minor G allele of this SNP reduces PCSK9 activity, as it is associated with lower cholesterolemia [10]. The serum lipid profile of a representative subset of the Badiangara cohort (*n* = 304) was recently examined in relation with common PCSK9 SNPs [11]. It was observed that, while the levels of total cholesterol and low-density lipoprotein-cholesterol (LDL-C) were similar between carriers and noncarriers of the G allele of the rs562556 SNP, the level of high-density lipoprotein-cholesterol (HDL-C) was significantly higher and that of triglycerides (TG) was significantly lower in carriers, resulting in lower TC/HDL and TG/HDL ratios. These results indicate that G allele carriers exhibit less dyslipidemia and, most probably, reduced severity of the disease, since dyslipidemia is a risk factor for malaria severity in mice [12] and humans [13].

Elevated plasma TG and inflammation are hallmarks of infectious diseases [14]. Exacerbation of the inflammatory response associates with bad prognosis in severe malaria [15]. A previous study of the Bandiagara cohort has shown that production of inflammatory cytokines is elevated in severe malaria cases relative to uncomplicated malaria cases or healthy controls [5]. In circulation, the level of PCSK9 correlates with those of glycerides [16] and proinflammatory cytokines [17]; on the other hand, HDL-c is known to possess anti-inflammatory properties [18]. We therefore propose that expression of the relatively inactive PCSK9 variant specified by the G allele of the rs562556 SNP reduces the TG/HDL index which attenuates *Plasmodium*-caused systemic inflammation and improves prognosis in severe malaria. Studies are needed to establish whether and how PCSK9 potency influences the expression of cytokines and chemokines in this disease.

The immune response to malaria seems to be modulated by circulating hemozoin [19]. This crystalline structure is formed in *Plasmodium*-infected red blood cells (RBC) as a means of detoxifying the free heme that results from hemoglobin degradation; it is released in circulation following schizont rupture of the RBC and is taken up by leucocytes [20]. Incidentally, using this same Bandiagara cohort, Lyke et al. [4] have shown that the frequency of hemozoin-containing leukocytes positively correlated with malaria fatality. Given the implication of acyl-glycerols in hemozoin formation (reviewed in [21]), it should be interesting to examine whether the reduced plasma level of TG in G allele carriers is associated with lower frequency of intraleucocytic hemozoin, contributing to the lower fatality to severe malaria observed in these carriers.

The association of the G allele with abdominal pain remains intriguing. This nonlethal and often transient symptom in severe malaria could originate in the liver, spleen, gallbladder, or kidneys as these organs become inflamed following sequestration of parasite-infected erythrocytes in their microvasculature [22]. We speculate that expression of the LOF PCSK9^V474^ variant in some of these organs could lead to much presence of LDLR and other scavenger receptors at the surface of their cells resulting in more internalization and accumulation of cholesterol and parasite metabolites (e.g., glycosylphosphatidylinositol (GPI), hemozoin) that could cause localized inflammation and abdominal pain. Noteworthy is the fact that, in this Bandiagara cohort, anti-GPI antibodies were higher among severe malaria children who died compared to those who survived [23], raising the possibility of a greater LDLR-mediated clearance of circulating GPI antigen in the latter group.

The missense rs562556 SNP is located in a genomic coding region that has been found to be under evolutionary selection, based on the rates of nonsynonymous versus synonymous variations in the primate phylum [24]. The G allele in particular seems to be under recent positive selection in populations of African ancestry [25]. It is possible that infectious diseases, malaria above all [26], were among the selective pressures that led to the high frequency (0.203) of the G allele in these populations; i.e., the allele might have protected children against early mortality from the diseases.

Susceptibility to malaria is a multifactorial phenomenon. It depends on biological, environmental, demographic, and socioeconomic as well as healthcare factors [27]. This study focuses on a genetic factor, i.e., the rs562556 SNP of the PCSK9 gene, in light of the mounting evidence indicating that the activity of the encoded protein influences immunity in infectious diseases [2]. The two main weaknesses of this study are (1) the limited number of cases; (2) the lack of data on correlations between plasma PCSK9 and immunity biomarkers (e.g., cytokines) in association with severe malaria outcomes.

## 5. Conclusion

We have observed a significant association of the minor allele of the rs562556 PCSK9 SNP with reduced mortality in severe malaria. This observation needs corroboration by larger studies taking into consideration immune, lipid, and PCSK9 parameters. Such a corroboration will justify the use of dietary or medicinal anti-PCSK9 interventions to attenuate the clinical course of malaria and reduce its mortality.

## Figures and Tables

**Table 1:** Association of clinical symptoms with death and survival.

Symptoms	Death/survival				*P* ^a^
	Died (*N* = 16)		Survived (*N* = 191)		
	*n*	(%)	*n*	(%)	
Convulsion	13	(81.3)	99	(51.8)	**0.0343**
Seizure	8	(50.0)	73	(38.2)	0.4265
Prostration	5	(31.5)	20	(10.5)	**0.0296**
Lethargy	10	(62.5)	41	(21.5)	**0.0074**
Coma	8	(50.0)	37	(19.4)	**0.0089**
Resp. symptoms	10	(62.5)	90	(47.1)	0.3007
Respiratory distress	7	(43.8)	3	(1.6)	**<0.0001**
Abdominal pain	2	(12.5)	37	19.4)	0.7417
Vomit	10	(62.5)	96	(50.3	0.4383
Repeated vomit	5	(31.3)	11	(5.8)	**0.0038**
Diarrhea	7	(43.8)	56	(29.3)	0.2613
No food	4	(25.0)	19	(9.9)	0.0851
Hypoglycemia^b^	2	(12.5)	2	(1.0)	**0.0308**
Severe anemia^c^	7	(43.8)	21	(11.0)	**0.0019**
Dark urine	3	(18.8)	14	(7.3)	1.0000
Jaundice	1	(6.2)	12	(6.3)	1.0000
Dysurea	1	(6.2)	5	(2.6)	0.3867
Hyperparasitemia^d^	2	(12.5)	100	(52.5)	**0.0030**

^a^ *P* by Fisher's exact test. Significance was set at 0.05. Significant values are bolded. ^b^Glucose ≤40 mg/dL; ^c^hemoglobin ≤5 g/dL; ^d^parasitemia ≥500 000/*μ*L.

**Table 2:** Association of death/survival outcomes with rs562556 genotypes.

Disease outcome	Genotypes				RR (95% CI)	*P* ^a^
	AA		AG/GG			
	(*N* = 143)		(*N* = 64)			
	*n*	(%)	*n*	(%)
Death	15	(10.5)	1	(1.6)	6.713 (1.19–39.53)	**0.0251**

^a^ *P* by Fisher's exact test. Significance was set at 0.05.

**Table 3:** Association with symptoms of malaria severity with rs562556 genotypes.

Symptoms	Genotypes				*P* ^a^
	AA		AG/GG		
	(*N* = 143)		(*N* = 64)		
	*n*	(%)	*n*	(%)	
Convulsion	73	(51.0)	29	(45.3)	0.4566
Seizures	61	(36.7)	25	(30.5)	0.4446
Prostration	17	(11.9)	8	(12.5)	1.0000
Lethargy	38	(26.6)	13	(20.3)	0.3856
Coma	31	(21.7)	13	(20.3)	0.4612
Resp. symptoms	64	(44.8)	36	(56.3)	0.1351
Respiratory distress	7	(4.9)	3	(4.7)	1.0000
Abdominal pain	16	(11.2)	23	(35.9)	**<0.0001**
Vomit	69	(48.2)	37	(57.8)	0.2303
Repeated vomit	13	(9.1)	3	(4.7)	0.4002
Diarrhea	42	(30.1)	21	(32.8)	0.6272
No food	16	(11.2)	7	(10.9)	1.0000
Hypoglycemia^b^	4	(2.8)	0	(0.0)	0.3135
Severe anemia^c^	22	(14.3)	6	(9.4)	0.2792
Dark urine	11	(8.2)	6	(9.4)	0.7849
Jaundice	8	(5.6)	5	(7.8)	0.5458
Dysurea	6	(4.2)	0	(0.0)	0.1801
Hyperparasitemia^d^	67	(46.9)	35	(55.6)	0.2960

^a^ *P* by Fisher's exact test. Significance was set at 0.05. Significant values are bolded. ^b^Glucose ≤40 mg/dL; ^c^hemoglobin ≤5 g/dL; ^d^parasitemia ≥500 000/*μ*L.

## Data Availability

The data used to support the findings of this study are available from the corresponding author upon request.

## Supplementary Materials

Supplementary Materials The supplementary file describes the sequence context of the rs562556 PCSK9 SNP, the fluorogenic probes, and the PCR protocol. Supplementary Table 1S: comparisons between children who died and those who survived (panel A) and between noncarriers and carriers of the LOF G allele of the rs562556 PCSK9 SNP (panel B) in anthropometry, ethnicity, lifestyle, medication, blood biochemistry, and parasitology.
