# Supplementary material for: Association of the rs562556 PCSK9 Gene Polymorphism with Reduced Mortality in Severe Malaria among Malian Children
Source: Can J Infect Dis Med Microbiol. 2020 Sep 16;2020:9340480. doi: 10.1155/2020/9340480 (PMC7532394; doi:10.1155/2020/9340480)
Supplement: Supplementary Materials — The supplementary file describes the sequence context of the rs562556 PCSK9 SNP, the fluorogenic probes, and the PCR protocol. Supplementary Table 1S: comparisons between children who died and those who survived (panel A) and between noncarriers and carriers of the LOF G allele of the rs562556 PCSK9 SNP (panel B) in anthropometry, ethnicity, lifestyle, medication, blood biochemistry, and parasitology. [file 9340480.f1.zip › 9340480.f1/Fedoryak et al_CJIDMM_Supplementary Table S1.pdf]

SUPPLEMENTARY TABLE S1

A. Death vs survival comparison

PARAMETERS	DEATH/SURVIVAL		P [d]
	**Died (N = 16)**	**Survived (N = 191**	
Age (months), mean ± SD (range)	27.9 ± 24.7 (7-97)	38.8 ± 25.1 (1-124)	0.097
Gender: F/M, n (%)	9/7 (56.2/43.7)	86/105 (45.0/55.0)	0.443
Tribe: Dogon/others, n (%)	14/2 (87.5/12.5)	153/38 (80.1/19.9)	0.742
Antimalarial medication [a], n (%)	6 (37.5)	81 (42.4)	0.796
Other medication [b], n (%)	7 (43.8)	99 (51.8)	0.608
Anti-mosquito protection [c]	13 (81.3)	56 (87.5)	1.000
Glycemia (mg/dL), mean ± SD [e]	96.9 ± 38.3	123.0 ± 43.0	**0.014**
Hemoglobinemia (g/dL) , mean ± SD	5.9 ± 2.8	8.7 ± 2.5	**< 0.0001**
Total WBC x10^3 (#/µl), mean ± SD [e]	19.3 ± 11.8	13.8 ± 7.3	**0.005**
Parasitemia x10^3 (#/µl) , mean ± SD [e]	20.6 ± 15.3	21.8 ± 27.8	0.061

B Genotype comparison

PARAMETERS	GENOTYPES		P [d]
	**AA (N = 143)**	**AG/GG (N = 64)**	
Age (months), , mean ± SD (range)	38.4 ± 23.0 (1-111)	43.9 ± 28.9 (3-124)	**0.026**
Gender (F/M), n (%)	65/78 (45.5/54.5)	30/34 (46.9/53.1)	0.881
Tribe (Dogon/others), n (%)	114/29 (79.7/20.3)	55/9 (85.9/14.1)	0.335
Antimalarial medication [a], n (%)	63 (44.1)	24 (37.5)	0.447
Other medication [b], n (%)	69 (48.3)	37 (57.8)	0.230
Anti-mosquitos protection n, (%) [c]	121 (84.6)	56 (87.5)	0.673
Hemoglobinemia (g/dL), mean ± SD [e]	8.3 ± 2.7	8.2 ± 2.4	0.189
Glycemia (mg/dL), mean ± SD [e]	117.9 ± 39.8	127.9 ± 49.6	0.124
Total WBC x10^3 (#/µl), mean ± SD [e]	14.4 ± 7.8	13.8 ± 8.0	0.587
Parasitemia x10^3 (#/µl), mean ± SD	20.6 ± 30.9	21.1 ± 16.6	0.886

[a] Antimalarial medications included chloroquine (94%) and quinine (6%).

[b] Anti-mosquito protection included bednets (76.7%), coils (22.2%), sprays (1.1%)

[c] Other medications included paracetamol (68.3), aspirin, (25.7%), traditional medicine (4.0%), and others (6.1%).

[d] *P* by Ficher's Exact test for % or Student *t* test for means. Significance set at 0.05. Significant values are bolded.

[e] In healthy children of the initial cohort, the mean ± SD (range) was 99.1 ± 1.1 (17 – 171) mg/dL for glycemia, 10.6 ± 0.1 (6.2 – 14.7) g/dL for hemoglobinemia, and 11.8 ± 0.3 (3.8 – 36.8) $x10^3$ for total WBC.
