# Supplementary material for: Association of the rs562556 PCSK9 Gene Polymorphism with Reduced Mortality in Severe Malaria among Malian Children
Source: Can J Infect Dis Med Microbiol. 2020 Sep 16;2020:9340480. doi: 10.1155/2020/9340480 (PMC7532394; doi:10.1155/2020/9340480)
Supplement: Supplementary Materials — The supplementary file describes the sequence context of the rs562556 PCSK9 SNP, the fluorogenic probes, and the PCR protocol. Supplementary Table 1S: comparisons between children who died and those who survived (panel A) and between noncarriers and carriers of the LOF G allele of the rs562556 PCSK9 SNP (panel B) in anthropometry, ethnicity, lifestyle, medication, blood biochemistry, and parasitology. [file 9340480.f1.zip › 9340480.f1/Fedoryak et al_CJIDMM_Supplementary Text.pdf]

Supplementary Text

TaqMan Assay

The sequence context of the *PCSK9* exon 9 from which allele-specific fluorogenic probes were derived was: cggatggccacagcc**[a/g]**tcgcccgctgcgccc. Predesigned primers and fluorogenic probes for were purchased from Applied Biosystems (Etobicoke, ON). The probes carried a fluorochrome at their 5' end: VIC for the common allele probe and FAM for the minor one; they both carried a non-fluorescent quencher (NFQ) at their 3-end.

A typical PCR reaction mixture contained 2 μL of DNA sample, 1x FastStart TaqMan ProbeMaster Rox master mix (Roche, Laval, QC), .9 μM primers, and 0.2 μM fluorogenic probes The reaction was run for 50 cycles of denaturation (95˚C, 20 sec), annealing (optimized temperature, 2 sec), and elongation (72˚C, 20 sec).
